# Minimally Invasive, CT Neuronavigated Posterolateral Pedicle Screw Placement in Upper Cervical Spine: A Retrospective Accuracy and Safety Analysis

**DOI:** 10.3390/jcm15114373

**Published:** 2026-06-05

**Authors:** Piotr Stogowski, Stanisław Adamski, Jakub Wiśniewski, Mateusz Węclewicz, Oskar Liczbik, Patryk Kurlandt, Jan Czauderna, Jonasz Tempski, Mateusz Szczupak, Jacek Kobak, Wojciech Wasilewski, Wojciech Kloc

**Affiliations:** 1 Department of Neurosurgery, Nicolaus Copernicus Hospital in Gdańsk, 80-803 Gdańsk, Poland; pstogowski@copernicus.gda.pl (P.S.); sadamski@copernicus.gda.pl (S.A.); jwisniewski@copernicus.gda.pl (J.W.); oliczbik@copernicus.gda.pl (O.L.); pkurlandt@copernicus.gda.pl (P.K.); jczauderna@copernicus.gda.pl (J.C.); jtempski@copernicus.gda.pl (J.T.); wwasilewski@wss.gda.pl (W.W.); wk56rak@gmail.com (W.K.); 2 Department of Neurosurgery, Collegium Medicum in Bydgoszcz, Nicolaus Copernicus University in Toruń, 85-094 Bydgoszcz, Poland; mateusz.wenclewicz@gmail.com; 3 Department of Anaesthesiology and Intensive Therapy, Nicolaus Copernicus Hospital in Gdańsk, 80-803 Gdańsk, Poland; mszczupak@copernicus.gda.pl; 4 Department of Otolaryngology, Faculty of Medicine, Medical University of Gdańsk, 80-214 Gdańsk, Poland; 5 Department of Health, WSB Merito University in Gdańsk, 80-266 Gdańsk, Poland

**Keywords:** C1 fracture, C2 fracture, pedicle screw fixation, minimally invasive spine surgery, intraoperative navigation, cervical spine

## Abstract

**Background**: Fractures of the upper cervical spine are challenging to treat due to their proximity to critical neurovascular structures and the need for immediate, stable fixation. Open posterior fixation remains the standard but is associated with soft-tissue disruption and morbidity. Minimally invasive, navigation-assisted pedicle screw fixation represents a viable alternative for older populations, significantly reducing surgical morbidity and tissue trauma. The present study evaluates the accuracy, safety, and perioperative outcomes of minimally invasive navigated posterolateral C1–C2 fixation. **Methods**: We conducted a retrospective consecutive case review of 51 patients who underwent minimally invasive C1–C2 screw fixation between 2019 and 2024. All procedures were performed using intraoperative O-arm imaging and StealthStation S8 navigation. Screw placement accuracy was assessed using the Bredow modification of the Gertzbein–Robbins and Heary classifications. Perioperative data, including operative time, screw dimensions, radiation dose, complications, and hospital stay, were recorded. **Results**: Fifty-one patients were included in the study. A total of 212 screws were placed. According to Gertzbein–Robbins grading, 92.4% were Grade A, 6.6% were Grade B, and 1% were Grade C. According to Heary grading, 95% were Grade I and 5% were Grade III. No vertebral artery injuries, new neurological deficits, or intraoperative hardware failures occurred. The mean screw lengths were 33.2 mm (SD = 3.38 mm) (C1) and 32 mm (SD = 4.30 mm) (C2). The mean operative time was 128 min (SD = 52.95 min). The mean radiation dose was 629.16 mGy·cm^2^ (SD = 372.2 mGy·cm^2^). One superficial wound infection occurred. The median postoperative NRS was 4 (IQR: 4–5). The mean hospital stay was 4.21 (SD = 3.77) days. **Conclusions**: Our findings demonstrate that the presented approach for C1–C2 fixation is a highly accurate and safe alternative to open posterior fixation for upper cervical fractures.

## 1. Introduction

C1–C2 fractures are among the most common upper cervical spine injuries, occurring predominantly after high-energy trauma in younger patients or minor falls in elderly individuals with compromised bone quality [1,2]. The unique anatomical configuration of the atlantoaxial joint provides a wide range of motion, rendering it biomechanically vulnerable, particularly at the odontoid and C1 ring. Unstable fractures in this region carry a substantial risk of neurological compromise and require stabilization to prevent catastrophic outcomes [3].

Traditional posterior fixation techniques, such as transarticular screws or the Goel-Harms C1 lateral mass–C2 pedicle screw construct, have demonstrated excellent fusion rates and biomechanical stability [4,5]. However, these open approaches necessitate extensive midline exposure, leading to paraspinal muscle stripping, increased blood loss, postoperative pain, and prolonged recovery [6]. Moreover, detachment of muscles from the C2 spinous process can be a potential risk factor for postoperative kyphotic deformity [7,8]. The proximity of the vertebral artery and spinal cord imposes a narrow margin for error, as mispositioned screws may result in serious complications [6,9].

The advent of intraoperative 3D imaging and neuronavigation has enabled more precise screw trajectory planning and real-time verification, significantly improving the accuracy of instrumentation in the upper cervical spine [10,11]. Minimally invasive approaches to the upper cervical spine are designed to preserve the integrity of the posterior tension band, reduce soft tissue morbidity, and upholding the biomechanical stability characteristic of rigid screw–rod constructs [12,13]. Despite these theoretical benefits, the literature on minimally invasive, navigation-assisted fixation techniques in the upper cervical spine remains limited; most reports describe small series or mixed cervical levels.

The present study evaluates the accuracy and safety of minimally invasive posterolateral pedicle screw fixation performed using intraoperative O-arm navigation in a series of consecutive patients with acute upper cervical fractures.

We hypothesize that this approach achieves high screw placement accuracy with a low complication rate.

## 2. Materials and Methods

A retrospective consecutive case review was conducted on patient data from individuals who underwent minimally invasive posterolateral lateral mass–pedicle screw fixation at the C1–C2 level with neuronavigation between 2019 and 2024. Inclusion criteria comprised patients with traumatic C1–C2 instability or type II and III odontoid fractures. Fracture morphology was classified according to the Anderson–D’Alonzo system for odontoid fractures and Levine and Edwards classification for C2 pedicle fracture. Patients with tumors and infections were excluded from the study. The study was conducted in compliance with the principles of the Declaration of Helsinki. The Bioethics Committee of the District Medical Chamber in Gdańsk reviewed the study protocol and determined that formal bioethical approval was not required due to retrospective nature of the study. Informed consent was also waived, as the interventions constituted standard medical treatment and all data were analyzed anonymously. Patient received the standard of care dictated by institutional protocols; all clinical decisions were made independently of this research.

### 2.1. Surgical Technique

**Figure 1:**
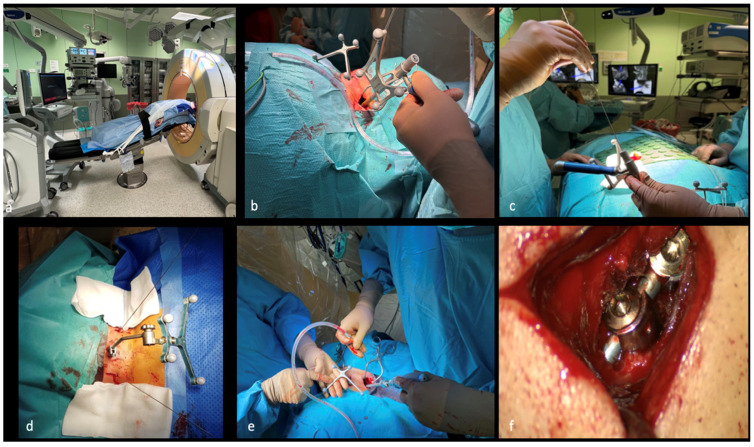
Illustration of minimally invasive posterolateral approach technique. (**a**) Intraoperative setting showing position of neuronavigation and O-arm; the patient is positioned in the Concorde position. (**b**) The reference frame is attached to the C2 spinous process; a cannulated, navigated probe was used to establish the proper K-wire trajectory. (**c**) Placement of the K-wire using a cannulated navigated instrument and a power drill to avoid applying torque on the bony structures. (**d**) K-wire placed in C2 pedicle. (**e**) The tap and pedicle screw were then advanced over the K-wire and into the bone. (**f**) Final view of the fixation within the posterolateral intermuscular corridor.

After general anesthesia, the patient’s head was fixed in a radiolucent carbon Mayfield clamp (DORO, Black Forest Medical GmbH, Frankfurt, Germany), and then the patient was placed in the Concorde position on a radiolucent operative table (Figure 1). All fractures were reduced using head maneuvers in the Mayfield clamp. This was either achieved by retro- or anteflexion depending on the fracture type. The correct anatomical alignment and reduction were controlled by fluoroscopy before draping. The patient was positioned through the gantry of the O-arm (Medtronic, Dublin, Ireland). A reference navigation frame was mounted to the C2 spinous process. To preserve muscle attachments, the reference array was affixed using a screw fixator through a small midline skin incision (Figure 1).

**Figure 2:**
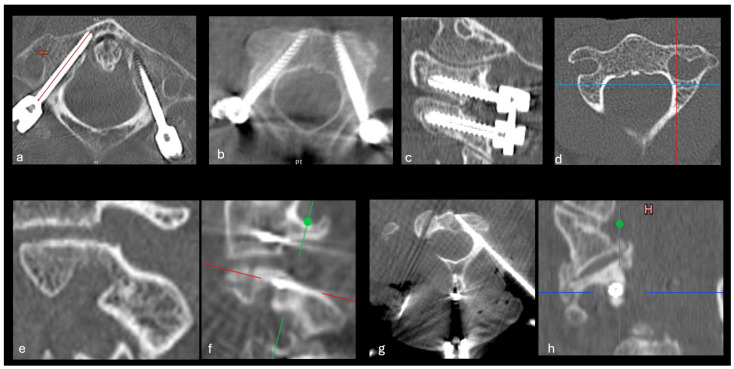
Clinical Imaging and grading analysis. (**a**–**c**) Intraoperative CT assessment (C1–C2): post-placement view of C1 and C2 screws following a posterolateral trajectory. The placement is classified as Gertzbein–Robbins Grade A (the screw is entirely contained within the pedicle/vertebral body with no cortical breach) and Heary Grade I (the screw tip is contained within the bone and does not violate the anterior vertebral wall). (**d**,**e**) Pedicle with high-riding vertebral artery: sagittal and axial views of a C2 pedicle in a patient with a high-riding vertebral artery. (**f**,**g**) K-wire trajectory HRVA: in these instances, the trajectory is adjusted to be more medially oriented and cephalad. This modified approach maximizes cortical bone purchase while ensuring that the screw remains safely clear of the vertebral artery’s path. (**h**) Intraoperative coronal view confirming successful screw placement within a narrow pedicle in the presence of an HRVA. The screw is positioned outside the transverse foramen, maintaining cortical integrity without violating the vessel.

This was followed by a 3D CT scan using the O-arm (Medtronic, Memphis, TN, USA). After image acquisition, the navigation system was initialized using the StealthStation S8 (Medtronic, Memphis, TN, USA). Navigation accuracy was initially verified using the C2 spinous process and mastoid tips as a landmark. A 3 cm skin incision was then made in the posterolateral neck region. A navigated Vertex Max trocar (Medtronic, Memphis, TN, USA) was passed to the facet–laminar part of C2 and the C1 mass. Using O-arm neuronavigated guidance, the screw trajectories were marked. Screws were planned intra-operatively in the navigation software regarding position and length. To protect the C2 nerve root, the entry point was established on the posterior arch of C1. We utilized a technique where the screw is placed partly through the posterior arch before entering the lateral mass, ensuring that the trajectory remains strictly superior to the C2 nerve root and venous plexus, thereby avoiding direct manipulation or tethering of the nerve. Subsequently, a second Kirschner wire (K-wire) was placed into C2 utilizing the standard pedicle trajectory. In cases with a high-riding vertebral artery or narrow pedicle isthmus, the trajectory was meticulously planned in the 3D navigation suite to optimize the corridor, typically directing the screw more medially and cephalad to maximize cortical purchase while avoiding the vessel. Placement was achieved using a cannulated, navigated instrument and a power drill to avoid generating torque on the bony structures. The position of the K-wire was confirmed with CT imaging to minimize the risk of navigation inaccuracy (Figure 1). K-wire positioning was corrected where required, followed by a repeat control CT scan. A self-tapping, cannulated pedicle screw was then advanced over the K-wire and into the bone. Also at this point, the size of the rod was determined in the planning software. After positioning the screws, a CT scan was performed to access screw placement. Intraoperative CT assessed and confirmed correct screw positioning, fracture reduction, and fixation of the fractured segment (Figure 2).

### 2.2. Assessment

For this study, the data sets were retrospectively analyzed by two independent examiners who were not involved in the surgical procedure. Each examiner performed the measurements twice. The intraobserver and interobserver agreement was measured. For the final analysis, the average of all four measurements was used. The position of the screws was graded according to the Bredow modification of the Gertzbein–Robbins and Heary classifications based on an intraoperative CT scan [14,15,16]. As the classifications are ordinal, “averaging” referred to the mode (most frequently assigned grade) across the four observations per screw; in the event of a tie, the more conservative (higher) grade was assigned.

### 2.3. Statistical Analysis

Data including screw length, operation time, radiation dose, intraoperative complications, postoperative pain measured using the Numeric Rating Scale (NRS), and mean hospital stay were collected. Cohen’s weighted kappa was used to calculate intra and inter-rater reliability.

The analysis was conducted using SPSS statistical software, version 26.0. Quantitative data were expressed as the mean and standard deviation (SD) or as the median and interquartile range (IQR) for NRS scores.

## 3. Results

**Figure 3:**
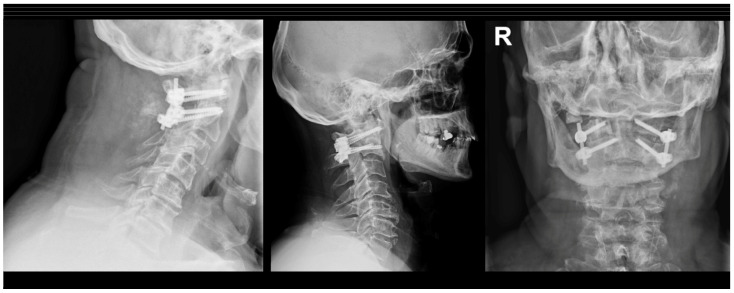
Postoperative standing X-rays after C1–C2 fusion.

A total of 51 patients were included in the study. Detailed demographic data are presented in Table 1. No hardware or software impairments were encountered. No intraoperative complications were observed. Intraoperative control scans from each patient were assessed according to the criteria described above. A total of 212 screws were placed and assessed by each investigator. Inter-rater reliability demonstrated nearly perfect agreement (Cohen’s κ = 0.89, 95% CI: 0.83–0.95, *p* < 0.001). Intra-rater reliability was similarly excellent, with Cohen’s κ = 0.91 (95% CI: 0.86–0.96, *p* < 0.001) for Rater 1 and κ = 0.93 (95% CI: 0.88–0.98, *p* < 0.001) for Rater 2. Of the 212 screws, 196 (92.4%) were rated Gertzbein–Robbins Grade A, 14 (6.6%) were rated Grade B, and two (0.9%) were rated Grade C; both Grade C screws were placed at C2. Using the Heary classification, the vast majority of screws—202/212 (95%)—were Grade I. Ten screws (5%) were classified as Grade III due to violation of the anterior vertebral body wall (Figure 2). Five K-wires in four patients required repositioning due to suboptimal trajectory identified on intraoperative CT prior to screw insertion. No final screw trajectories required intraoperative repositioning. No complications related to K-wire or screw position were identified; specifically, no vertebral artery violation and no new postoperative neurological deficit were recorded. Two patients experienced unilateral transient occipital neuralgia, which resolved following medical treatment. The mean blood loss was 257.55 (SD = 131.67) mL. All screws were 4 mm in diameter. The mean screw length was 33.2 (SD = 3.38) mm for C1 and 32.0 (SD = 4.30) mm for C2. The mean operative time was 128 ± 52.95 min. The mean radiation dose, measured as the dose–area product (DAP), was 629.16 (SD = 372.2) mGy·cm^2^. The median number of intraoperative scans per procedure was 3 (IQR: 3–3); additional scans were required in the four patients who needed K-wire repositioning. During the postoperative course, one superficial wound infection was observed, with a good outcome following treatment. The median Numeric Rating Scale (NRS) pain score on the first postoperative day was 4 (IQR: 4–5). Three patients required occasional opioid analgesia. The mean hospital stay was 4.21 (SD = 3.77) days. Postoperative standing X-rays after C1–C2 fusion are shown in Figure 3.

## 4. Discussion

Surgical management of fractures in the C1–C2 region remains challenging due to the complex anatomy and serious complications. This retrospective study suggests that C1–C2 posterior fixation using a minimally invasive posterolateral approach with neuronavigation is a highly accurate and safe surgical technique. Our results demonstrate a high rate of optimal screw placement, with 92.4% (196/212) of screws achieving a Gertzbein–Robbins Grade A rating and 95% (202/212) were classified as Heary Grade I. The anatomical complexities of the atlantoaxial spine present significant challenges to surgical fixation. The proximity of the vertebral arteries, spinal cord, and C2 nerve root makes freehand screw placement a technically demanding procedure with a recognized risk of complications and a steep learning curve [3]. A recent meta-analysis reported vertebral artery injury rates ranging from 2.2% to 3.5%, with some series reach up to 8% [17,18]. Malposition rates for C2 pedicle screws have been reported to be up to 16% [19]. In our series, there were no cases of vertebral artery violation or new neurological deficit. The high accuracy observed in our series is consistent with the results of other studies employing navigation for C1–C2 fixation [13]. Our findings are in agreement with past reports, further validating the technique as a promising alternative to traditional freehand methods [10,11,12,13,20]. Despite initial concerns regarding the transition to a new operative method, the MISS approach proved to be ergonomically favorable and highly satisfying for the operating surgeons. The use of neuronavigation significantly enhanced surgeon comfort and confidence when operating in the atlantoaxial region. This was particularly valuable for surgeons without prior experience in C1–C2 fixation. Despite limited prior exposure, surgeons demonstrated rapid acquisition of proficiency in C1–C2 fixation, achieving safe operative outcomes after a short initial learning phase.

Subsequent studies have shown that minimally invasive adaptations can reduce soft-tissue trauma, blood loss, and recovery time without compromising stability [21,22]. By minimizing muscle dissection, this approach contributed to reduced blood loss and rapid postoperative recovery, as evidenced by a mean hospital stay of 4.21 days and a median pain score of 4 on the NRS on the first postoperative day. These factors are particularly relevant in elderly or comorbid trauma patients, as they represent a significant portion of C1–C2 trauma cases. Furthermore, our posterolateral approach with neuronavigation allows for the precise and repeatable placement of longer and more anatomically optimized screws. The C2 pedicle screw trajectory, which is planned and executed with image guidance, is more cephalad and medial than what is typically achievable with a traditional open midline approach. This trajectory allows the screw to traverse a greater length of dense cortical bone in the C2 pedicle and vertebral body, as reflected by our mean C2 screw length of 32 mm. Biomechanical studies have consistently shown that a longer bone-screw interface provides superior pullout strength and biomechanical stability compared to shorter screws [23]. This enhanced screw purchase is critical for achieving a robust fixation construct and a high fusion rate, even in patients with compromised bone quality and a high-riding vertebral artery. While the traditional open pars screw approach can be effective, its entry point and trajectory are often more limited, which may restrict the achievable screw length and purchase within the C2 body [24]. Furthermore, in patients with HRVA, we utilized the navigation software to plan a specific “superomedial” trajectory that maximizes cortical purchase in the upper portion of the pedicle, avoiding the vertebral artery loop (Figure 2). Our screw placement accuracy—92.4% Grade A according to the Gertzbein–Robbins classification and 95% Grade I according to the Heary system—aligns with the rates reported in the navigated upper cervical fixation literature, where Grade A accuracy typically ranges from 85% to 95% [9,12,24,25,26]. The absence of vertebral artery injury in our series is notable. This likely reflects the combined benefits of meticulous preoperative CT angiographic planning, intraoperative O-arm imaging, and real-time navigation. The mean operative time of 128 min was comparable to that reported in freehand series [18]. The upfront time investment for patient registration and intraoperative CT scanning is offset by the elimination of repetitive fluoroscopic checks [18]. However, with freehand techniques, multiple fluoroscopic images and screw adjustments are often necessary to confirm correct trajectory, thereby increasing both operative time and radiation exposure to the patient and surgical team. Furthermore, the high initial accuracy facilitated by navigation contributes to reduced revision rates and avoids the morbidity associated with screw malposition [27].

Intraoperative O-arm navigation is the reason for the associated radiation exposure. Our mean radiation dose of 629.16 mGy-cm^2^ is comparable to values reported in other navigated spine studies [27]. In our study, a median of three scans per case resulted in a non-trivial cumulative dose, requiring surgeons to carefully balance the risks of radiation exposure against the benefits of enhanced screw accuracy. However, recent advances in imaging technology, such as low-dose Smart Dose protocols, have demonstrated the potential to significantly reduce the total radiation burden without compromising the visualization quality necessary for precise navigation. Future integration of these low-dose protocols could further mitigate this drawback while maintaining the safety profile of the procedure. The enhanced patient safety and reduced risk of catastrophic complications arguably compensate for the risks of radiation exposure. To minimize the surgical team’s exposure, members maintained a safe distance from the O-arm gantry during image acquisition. No surgical staff remained within the primary beam during scanning.

A critical technical limitation inherent to neuronavigation is the absolute dependence on the stability of the reference frame. The entire navigation system relies on a precise, unmoving relationship between the patient’s anatomy and the reference array attached to it. Any unintended movement of the reference frame during the procedure, even by a few millimeters, will introduce a registration error or image-to-patient mismatch [28]. This can lead to a false sense of security, as the surgeon’s navigated instruments may appear to be in the correct position on the screen when, in reality, they are off-target. This risk of inaccurate trajectory is a significant concern and may potentially lead to catastrophic neurovascular injury [29,30]. To mitigate this, the reference array was securely fixed to the C2 spinous process with a screw fixator, and accuracy was routinely verified intraoperatively against known anatomical landmarks, including the external occipital protuberance, mastoid process, and spinous process of C2. Furthermore, to compensate for the lack of anatomical exposure during percutaneous navigation, K-wire positioning was verified via intraoperative CT. This step serves as a critical checkpoint to validate the navigation system’s precision prior to final screw insertion. The accuracy of this approach was demonstrated by the low revision rate: only five K-wires in four patients required intraoperative adjustment—none of which involved neurovascular conflict—and no final screw trajectories necessitated repositioning.

### Limitations

Our study is not without limitations. As a single-center retrospective review, it is susceptible to selection bias. The absence of a control group (i.e., a freehand cohort) prevents a direct, head-to-head comparison of outcomes, though the literature provides a robust basis for comparison. The sample size, while reasonable for this specific type of surgery, is limited. Therefore, this study should be regarded as a preliminary description of a surgical technique with evaluations of accuracy and safety. Consequently, long-term data regarding fusion rates, hardware failure and late biomechanical outcomes are not currently available. Patient-reported functional outcomes (NDI, VAS neck pain, and SF-36) were not systematically collected in this retrospective cohort. A prospective cohort study to evaluate these long-term parameters is ongoing. Furthermore, the described technique avoids the use of a C1–C2 lateral joint bony graft, which limits lateral joint fusion. The investigators are presently conducting a prospective cohort study to rigorously assess the long-term functional and radiographic outcomes of this approach, specifically examining the true clinical effect and the rate of fusion in the lateral C1–C2 joint. Prospective, multicenter studies with larger cohorts and long-term follow-up are warranted to validate these findings, assess fusion rates, and evaluate patient-reported outcomes. Comparative cost-effectiveness analyses between minimally invasive and open C1–C2 fixation could further inform surgical decision-making. The integration of robotic guidance systems may offer additional precision and warrants investigation. These limitations mean that the present study provides evidence of technical feasibility and perioperative safety, but not of long-term efficacy.

## 5. Conclusions

Minimally invasive navigated C1–C2 fixation appears feasible and safe, with high screw placement accuracy in this retrospective series. Prospective comparative studies with long-term fusion outcomes are required to establish clinical effectiveness. The potential for muscle preservation and precise screw placement makes it an interesting alternative to open techniques, particularly for cases with aberrant anatomy and elderly patients.

## Figures and Tables

**Table 1:** Patients’ general information and fracture characteristics.

Age	72.5 (SD = 8.98) Years
Gender	
Male	28
Female	23
**Type of injury**	
C1–C2 instability	11
C2 type II Anderson D’Alonzo	24
C2 type III Anderson D’Alonzo	12
C2 type II/III + C2 Levine and Edwards type III	4
**Instrumented segment**	**Patients/Screws**
C1–C2	47/188 screws
C1–C2–C3	4/24 screws

## Data Availability

The original contributions presented in this study are included in the article. Further inquiries can be directed to the corresponding authors.

## Abbreviations

The following abbreviations are used in this manuscript:

DAP	dose area product
HRVA	high-riding vertebral artery
NRS	Numeric Rating Scale
MISS	minimally invasive spine surgery
IQR	interquartile range
SD	standard deviation
